# Moderate-intensity exercise alleviates pyroptosis by promoting autophagy in osteoarthritis via the P2X7/AMPK/mTOR axis

**DOI:** 10.1038/s41420-021-00746-z

**Published:** 2021-11-10

**Authors:** Zihao Li, Ziyu Huang, He Zhang, Jinghan Lu, Yicheng Tian, Shang Piao, Zhiming Lin, Lunhao Bai

**Affiliations:** 1 Department of Orthopedics, Shengjing Hospital of China Medical University, Shenyang, 110024 China; 2 Foreign Languages College, Shanghai Normal University, Shanghai, 200234 China

**Keywords:** Autophagy, Cell death

## Abstract

Instability and excessive use of the knee joint can cause osteoarthritis (OA). Reasonable exercise can enhance the stability of the knee joint and prevent and relieve the occurrence and development of OA. As a key switch for inflammation, P2X purinoceptor 7 (P2X7) has attracted much attention in studies of OA. Exercise can regulate P2X7 expression and activation. However, the role of P2X7 in exercise-based prevention and treatment of OA is unknown. We previously showed that moderate-intensity exercise can significantly alleviate OA symptoms. Accordingly, in this study, we evaluated the effects of exercise on P2X7 expression and activation in chondrocytes. Micro-computed tomography, hematoxylin, and eosin staining, Toluidine Blue O staining, immunohistochemistry, and terminal deoxynucleotidyl transferase dUTP nick-end labeling experiments showed that P2X7 expression was lower in the moderate-intensity exercise group than in the inflammation and low- and high-intensity exercise groups. Additionally, chondrocyte death, cartilage destruction, and the degree and severity of pyroptosis were significantly reduced, whereas autophagy levels were significantly increased in the moderate-intensity exercise group. Cell Counting Kit-8 assay, lactate dehydrogenase release, flow cytometry, enzyme-linked immunosorbent assay, cell fluorescence, western blot, reverse transcription-quantitative polymerase chain reaction, and transmission electron microscopy experiments showed that moderate activation of P2X7 promoted autophagy through the AMP-activated protein kinase (AMPK)/mammalian target of rapamycin (mTOR) signaling pathway and promoted autolysosome targeting for degradation of the inflammasome component NLRP3, thereby inhibiting pyroptosis. Additionally, the use of AMPK and mTOR activators and inhibitors indicated that the AMPK-mTOR signaling pathway, as the downstream of P2X7, played a key role in delaying the occurrence and development of OA. We propose that moderate-intensity exercise promoted chondrocyte autophagy through the P2X7/AMPK/mTOR signal axis to alleviate pyroptosis. Our findings provide novel insights into the positive and preventative effects of exercise on OA.

## Introduction

The knee is essential to movement, is more flexible, and more prone to injury. Osteoarthritis (OA) causes physical pain and even disability in patients and results in increased socio-economic costs. Identification of effective treatments for OA is necessary to improve public health worldwide [1].

P2X purinoceptor 7 (P2X7) is a purinergic receptor and trimeric adenosine triphosphate (ATP)-gated cation channel that is expressed in several types of eukaryotic cells, including immune and bone cells. Moderate activation occurs when the receptor binds to ATP. When the gated state of P2X7R is open, the receptor mediates Na^+^ and Ca^2+^ influx and K^+^ efflux, resulting in rapid depolarization [2]. When the activation time is prolonged, P2X7R can induce membrane pore formation, allowing molecules up to hundreds of Da to pass [3]. As a key inflammatory switch, the activation of P2X7R mediates several downstream reactions, such as inflammatory factor release, cell proliferation, cell death, and phenotypic changes [4]. K^+^ efflux is one of the factors for activation of the NLRP3 inflammasome [5]. Indeed, when cells undergo pyroptosis, cell swelling and rupture are accompanied by the uncontrolled release of many pro-inflammatory cytokines, such as interleukin (IL)-1β, leading to passive and rapid cell death [6]. IL-1β activated by caspase-1 cleavage not only induces apoptosis [7] but also stimulates the secretion of cartilage-degrading enzymes, such as matrix metalloproteinase (MMP) 3, MMP13, and a disintegrin and metalloproteinase with thrombospondin motifs-4 and -5 [8, 9], leading to the degradation of type II collagen and proteoglycan in the extracellular matrix and aggravating the occurrence and development of OA.

In addition, P2X7-mediated Ca^2+^ influx [10, 11] and increased AMP/ATP ratios can activate the energy receptor AMP-activated protein kinase (AMPK), inhibits mammalian target of rapamycin (mTOR), and promote autophagy [12]. Cells recover excess or damaged lipids, proteins, and organelles through autophagy; these proteins are then degraded and reused to maintain cell viability and relieve inflammation [9, 13]. Rapamycin (an autophagy inducer) promotes the degradation and clearance of damaged mitochondria, reduces IL-1β-induced reactive oxygen species (ROS) generation, and blocks the OA-like phenotype in chondrocytes [14]. mTOR overexpression inhibits chondrocyte autophagy and promotes apoptosis, leading to increased cartilage degeneration [15]. However, studies have shown that the activation state of P2X7 affects the level of autophagy in cells [16, 17]. In the early stage of activation, P2X7-mediated K^+^ efflux and Ca^2+^ influx activate mitochondrial ROS (mtROS), and Ca^2+^ activates Ca^2+^/calmodulin-dependent protein kinase, which in turn activates the AMPK pathway, inhibits mTOR, and promotes mitophagy and lysosome biogenesis. During the later stages of activation, the lysosome acts as a signaling hub between AMPK and mTOR; its stability is destroyed, and it cannot be fused with autophagosomes to form autolysosomes for targeting and degrading harmful substances, such as inflammasome components, leading to apoptosis, pyroptosis [18], and ultimately cell death [19]. Based on the above research results, we speculate that modulation of the ion current by variations in the expression and activation of P2X7 can cause changes in the fate of cell autophagy and pyroptosis. When the chondrocyte catabolism balance is disrupted, cartilage degradation is intensified, which leads to the aggravation of OA.

Although our previous work initially explored the connection between P2X7 and OA [20, 21], the role of P2X7 in exercise prevention and treatment of OA is still unknown. P2X7 modulates IL-1β levels in the plasma after exercise, and the expression of P2X7 increases with the duration of exercise. After exercise, the levels of P2X7R and nuclear factor-κB decrease with the duration of exercise. In sedentary individuals, the levels of NLRP3 and caspase-1 increase after exercise; in contrast, NLRP3 levels decrease after exercise in individuals participating in normal or endurance exercise. Thus, exercise exerts pro-inflammatory effects in sedentary individuals and anti-inflammatory effects in individuals who exercise [22].

Therefore, in this study, we evaluated whether moderate-intensity exercise could promote autophagy and inhibit pyroptosis through the P2X7/AMPK/mTOR axis, thereby alleviating the occurrence and development of OA. Our findings are expected to help elucidate the role of P2X7 in OA and contribute to the development of reasonable and appropriate exercise regimens to prevent and treat OA.

## Results

### Moderate-intensity exercise maintained low activation of P2X7, promoted autophagy, and inhibited pyroptosis

**Fig. 1:**
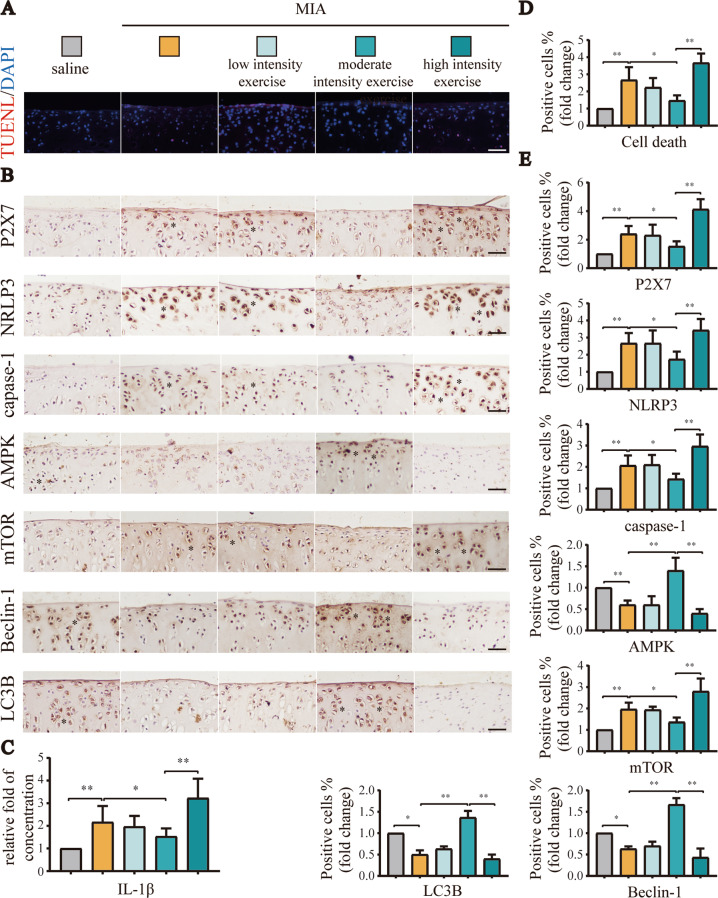
Moderate-intensity exercise downregulated P2X7, promoted autophagy, and inhibited pyroptosis. We set the following five groups: saline, MIA, MIA + low-intensity exercise, MIA + moderate-intensity exercise, MIA + high-intensity exercise. A total of 50 rats were randomly assigned, with 10 rats in each group. The animal tissues in this part, such as paraffin sections and joint cavity lavage fluid, came from previous experiments of our research group [23]. **A** TUNEL assays were used to evaluate cell death in tissue sections from each group. Red: dead cells; blue: nuclei stained by DAPI (scale bar: 50 μm). **B** IHC was used to evaluate P2X7, NLRP3, caspase-1, mTOR, AMPK, LC3B, and Beclin-1 expression in tissue sections from each group. Brown: stained cells; blue: hematoxylin-stained nuclei (scale bar: 500 μm). **C** ELISA were used to detect IL-1β content in the articular cavity lavage fluid from each group. **D** Statistical data for cell death in TUNEL assays. **E** Statistical data for stained cells in IHC analysis. The * in the histochemical images represents the positively stained cells. Data are presented as means ± standard deviations of at least three independent experiments. *p < 0.05, **p < 0.01.

Our previous research work showed that moderate-intensity exercise reduced the occurrence and development of OA [23]. Disorders of the chondrocyte state balance, such as cell death, are the main factors aggravating OA. The animal tissues in this part, such as paraffin sections and joint cavity lavage fluid, came from previous experiments of our research group [23]. In this study, TUNEL assays (Fig. 1A, D) showed that moderate-intensity exercise significantly reduced cell death in the knee joint tissue of OA model rats compared with low- and high-intensity exercise. The unmerged images are shown in Fig. S3A. As a key switch for inflammation, P2X7 is closely related to cell death. IHC analysis (Fig. 1B, E) showed that P2X7 expression first decreased and then increased as the exercise intensity increased, with lower expression observed in the moderate-intensity exercise group. Moreover, the expression levels of pyroptosis-related indicators, such as NLRP3 and caspase-1, also decreased in the moderate-intensity exercise group. By contrast, autophagy-related indicators, such as LC3B and Beclin-1 were upregulated, and changes in the expression levels of AMPK/mTOR pathway indicators also confirmed that moderate-intensity exercise promoted autophagy. ELISAs (Fig. 1C) showed that IL-1β levels in IALF initially decreased and then increased as the exercise intensity increased. Based on the above results, we concluded that moderate-intensity exercise downregulated P2X7, promoted autophagy, and inhibited pyroptosis.

### Moderate-intensity exercise inhibited pyroptosis and alleviated OA by moderately activating P2X7-mediated autophagy

**Fig. 2:**
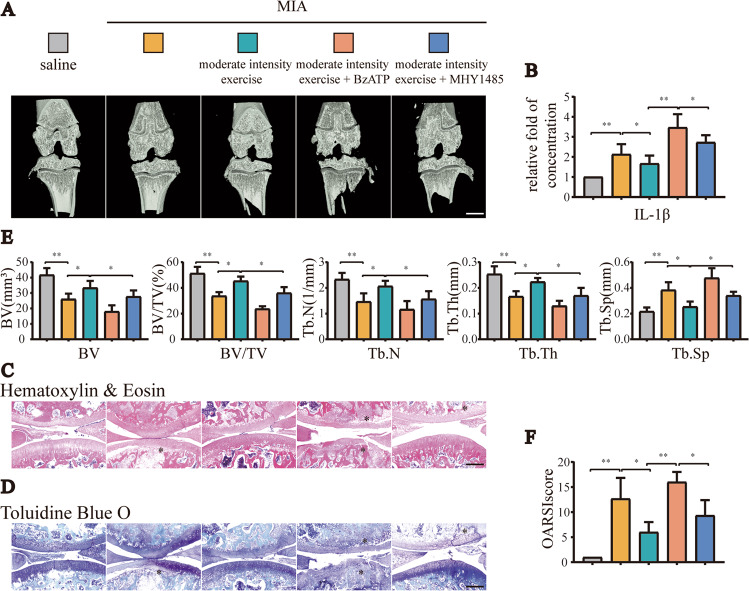
Increased expression of P2X7 or inhibition of autophagy offset the anti-inflammatory effects of moderate-intensity exercise. We set the following five groups: saline, MIA, MIA + moderate-intensity exercise, MIA + moderate-intensity exercise + BzATP, MIA + moderate-intensity exercise + MHY1485. A total of 50 rats were randomly assigned, with 10 rats in each group. **A** Micro-CT scanning of knee joints in each group was used to obtain imaging data for the tibial plateau and subchondral bone (scale bar: 1 mm). **B** ELISA was used to determine IL-1β levels in the articular cavity lavage fluid of rats in each group. **C** H&E staining and **D** TB staining were used to analyze the degree of cartilage loss and the development of inflammation. **E** Knee joint bone-related parameters, including BV, BV/TV, Tb.N, Tb.Th, and Tb.Sp, as evaluated by micro-CT. **F** OARSI scores were used to determine the development stage of OA in the knee joint tissues of rats in each group. The * in the HE and TB staining images represents the cartilage damage site. Data are presented as means ± standard deviations of at least three independent experiments. *p < 0.05, **p < 0.01.

In addition to the intervention of treadmill exercise in OA model rats, we also injected the P2X7 agonist BzATP (0.3 mg) or the autophagy inhibitor MHY1485 (0.3 mg) into the knee joint cavity of rats to explore whether the anti-inflammatory effects exerted by moderate-intensity exercise functioned via maintaining the appropriate activation of P2X7 and promoting autophagy. The results of micro-CT analysis (Fig. 2A, E) showed that bone destruction in rats in the OA group injected with MIA into the knee joint cavity was severe, and treadmill training with moderate intensity reduced osteophyte formation and joint space narrowing. In the group injected with BzATP or MHY1485, the anti-inflammatory effects of exercise disappeared, and even more serious OA symptoms appeared. The results of ELISA (Fig. 2B) showed that IL-1β levels in the IALF decreased after moderate-intensity exercise, and this trend was reversed after the additional injection of BzATP or MHY1485. The results of H&E and TB staining (Fig. 2C, D) showed that moderate-intensity exercise reduced cartilage loss and alleviated the occurrence and development of OA (Fig. 2F). BzATP upregulated P2X7, and MHY1485 inhibited autophagy; thus, the anti-inflammatory effects were blocked.

**Fig. 3:**
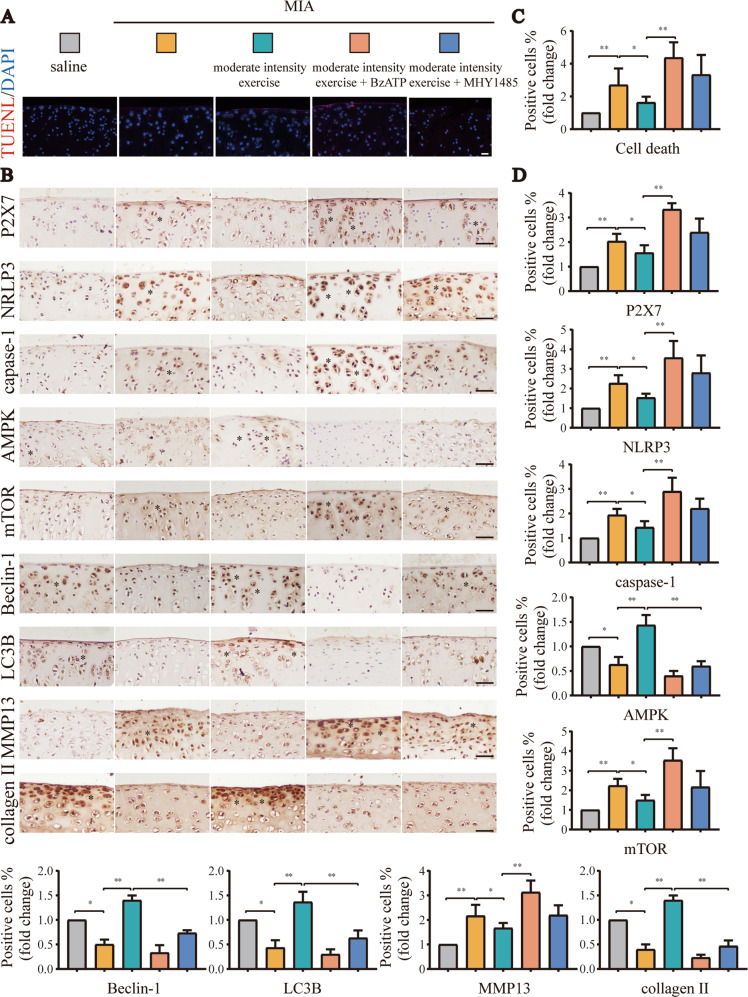
Supplement to Fig. 2. **A** TUNEL assays were used to detect cell death in tissue sections from each group. Red: dead cells; blue: nuclei stained by DAPI (scale bar: 50 μm). **B** IHC was used to detect the expression levels of P2X7, NLRP3, caspase-1, mTOR, AMPK, LC3B, and Beclin-1 in tissue sections from each group. Brown: stained cells; blue: hematoxylin-stained nuclei (scale bar: 500 μm). **C** Statistical data for cell death in TUNEL assays. **D** Statistical data for stained cells in IHC analyses. The * in the histochemical images represents the positively stained cells. Data are presented as means ± standard deviations of at least three independent experiments. *p < 0.05, **p < 0.01.

Similar results were observed in TUNEL assays (Fig. 3A) and IHC analyses (Fig. 3B). The unmerged pictures are shown in Fig. S3B. Additional injection of BzATP significantly increased P2X7 expression, accompanied by the aggravation of pyroptosis and decreased autophagy, resulting in increased numbers of dead cells (Fig. 3C). Additionally, MHY1485 reduced the expression levels of autophagy-related indicators, leading to increased cell death and more obvious inflammation (Fig. 3D). Based on the above results, we inferred that moderate-intensity exercise inhibited pyroptosis and alleviated OA by maintaining the appropriate activation of P2X7 and promoting autophagy. Accordingly, overactivation of P2X7 or inhibition of autophagy blocked the anti-inflammatory effects of moderate-intensity exercise.

### BzATP moderately activated P2X7, promoted autophagy, and inhibited pyroptosis, and excessive activation of P2X7 aggravated OA

**Fig. 4:**
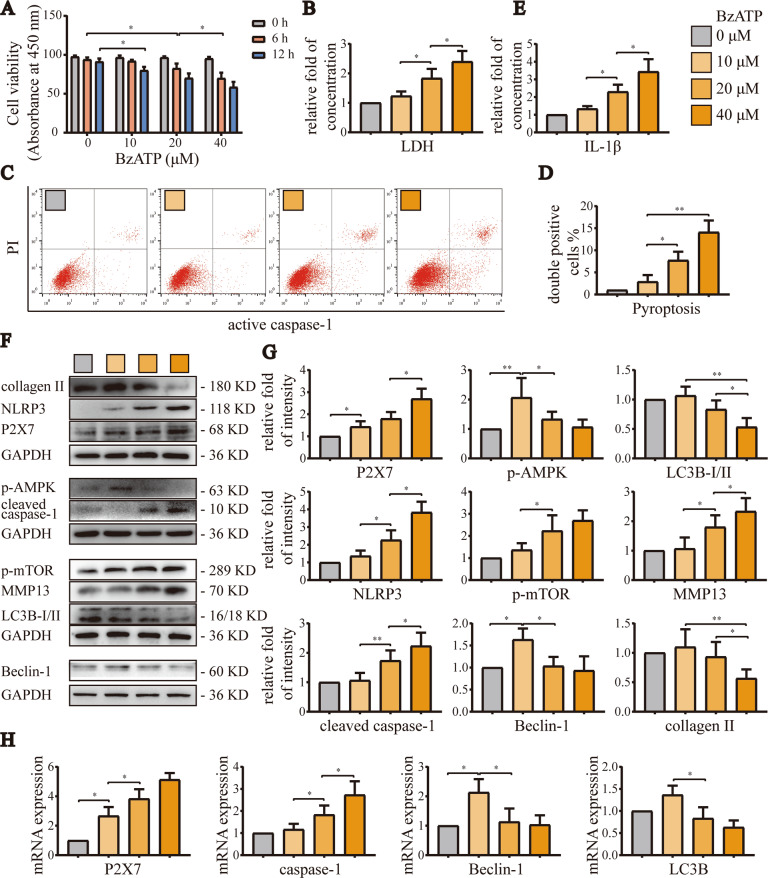
Activation of P2X7 with increasing concentrations of BzATP first promoted autophagy and then induced pyroptosis. **A** CCK-8 assays were used to evaluate cell viability in each group. Absorbance was measured at a wavelength of 450 nm. **B** LDH release assays were used to detect the degree of cell damage. **C** Flow cytometry was used to detect the number and ratio of caspase-1/PI-stained cells, reflecting the severity of cell pyroptosis. **D** Statistical data for stained cells. **E** ELISA was used to detect IL-1β levels in cell culture supernatants from each group. **F**, **G** Western blotting and **H** RT-qPCR were used to detect protein and mRNA expression levels of P2X7, NLRP3, caspase-1, mTOR, AMPK, LC3B, Beclin-1, MMP13, and collagen II. Data are presented as means ± standard deviations of at least three independent experiments. *p < 0.05, **p < 0.01.

In order to determine the appropriate concentration and treatment time for BzATP-induced P2X7 activation, we evaluated cell viability for various BzATP concentrations (0, 10, 20, and 40 μM) and treatment times (12 and 24 h). CCK-8 assays (Fig. 4A) showed that cell viability was significantly reduced by BzATP treatment for 6 h at a concentration of 20 μM. Moreover, when the BzATP concentration was 40 μM, the cell viability dropped to 70.1% that of the control group, and after treatment for 12 h, cell viability decreased significantly when the concentration of BzATP was 10 μM. Therefore, for subsequent cellular experiments, we treated cells with BzATP for 6 h.

LDH release experiments (Fig. 4B) and flow cytometry analysis (Fig. 4C, D) showed that when the concentration of BzATP was 10 μM, pyroptosis was not induced. However, as the concentration increased, cell death and pyroptosis became more obvious. Similarly, ELISA (Fig. 4E) showed that the IL-1β content in cell culture supernatants was not significantly different from the control group when the BzATP concentration was 10 μM. However, when the concentration of BzATP was increased, IL-1β levels in cell culture supernatants increased significantly.

Western blot analysis (Fig. 4F, G) and RT-qPCR (Fig. 4H) also confirmed the above results, demonstrating that P2X7 mRNA and protein levels were increased as the BzATP concentration increased. Indeed, 10 μM BzATP moderately activated P2X7 and increased the expression levels of autophagy-related targets, while decreasing the expression levels of pyroptosis-related targets. At 20 and 40 μM, BzATP overactivated P2X7, inducing the synthesis of MMP13 and the degradation of collagen II, leading to the opposite phenomena and worsening the inflammatory response. Based on the above results, we inferred that BzATP moderately activated P2X7, promoted autophagy, inhibited pyroptosis, restored cell viability, reduced cell death, and alleviated OA. By contrast, excessive activation of P2X7 promoted pyroptosis, inhibited autophagy, and aggravated OA.

### Excessive activation of P2X7 aggravated the occurrence and development of OA by inducing pyroptosis

**Fig. 5:**
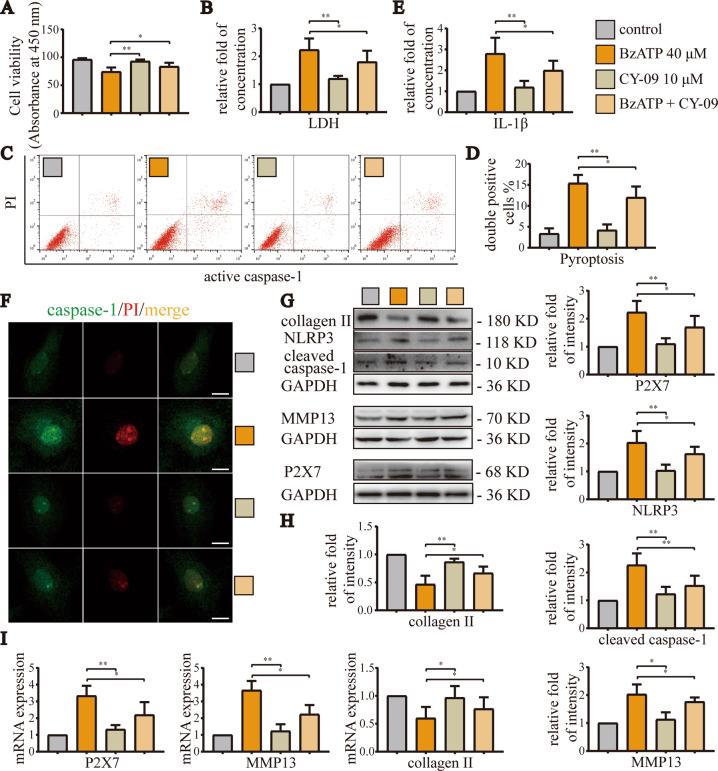
Inhibition of NLRP3 alleviated OA caused by overactivation of P2X7. **A** CCK-8 assays were used to detect cell viability. Absorbance was measured at a wavelength of 450 nm. **B** LDH release assays were used to detect the degree of cell damage. **C** Flow cytometry was used to detect the number and ratio of caspase-1/PI-stained cells, reflecting the severity of cell pyroptosis. **D** Statistical data for stained cells. **E** ELISA was used to detect IL-1β levels in cell culture supernatants for each group. **F** Cell fluorescence experiments were used to determine the fluorescence intensity and location of caspase-1/PI staining in cells, reflecting the severity of cell damage and pyroptosis (scale bar: 10 μm). **G**, **H** Western blotting and **I** RT-qPCR were used to detect the protein and mRNA expression levels of P2X7, NLRP3, caspase-1, MMP13, and collagen II. Data are presented as means ± standard deviations of at least three independent experiments. *p < 0.05, **p < 0.01.

In order to explore whether high-level activation of P2X7 induced pyroptosis to aggravate OA, we selected the NLRP3 inhibitor CY-09 for subsequent cell experiments. The results of CCK-8 assays (Fig. 5A) showed that CY-09 (10 μM) alone did not cause significant changes in cell viability, whereas BzATP (40 μM) significantly reduced cell viability. After treatment with CY-09, cell viability was restored. The results of LDH release assays (Fig. 5B) and flow cytometry (Fig. 5C, D) showed that cell damage and pyroptosis caused by BzATP were alleviated after application of the NLRP3 inhibitor. Additionally, ELISA (Fig. 5E) showed that CY-09 effectively reduced IL-1β levels in the cell culture supernatant, indicating that the inflammatory response was reduced. Cell fluorescence analyses (Fig. 5F) showed that the fluorescence intensity of caspase-1 and PI double staining decreased significantly after inhibition of NLRP3. Western blotting (Fig. 5G, H) and RT-qPCR (Fig. 5I) confirmed the above results. Moreover, CY-09 reduced the expression levels of P2X7 and pyroptosis-related indicators, blocked the synthesis of MMP13, and inhibited the degradation of collagen II. Based on these results, we concluded that excessive activation of P2X7 aggravated the occurrence and development of OA by inducing pyroptosis. Inhibition of NLRP3 blocked pyroptosis and cell death, thereby alleviating OA.

### Moderate activation of P2X7 induced autophagy through the AMPK/mTOR signaling pathway to block pyroptosis

**Fig. 6:**
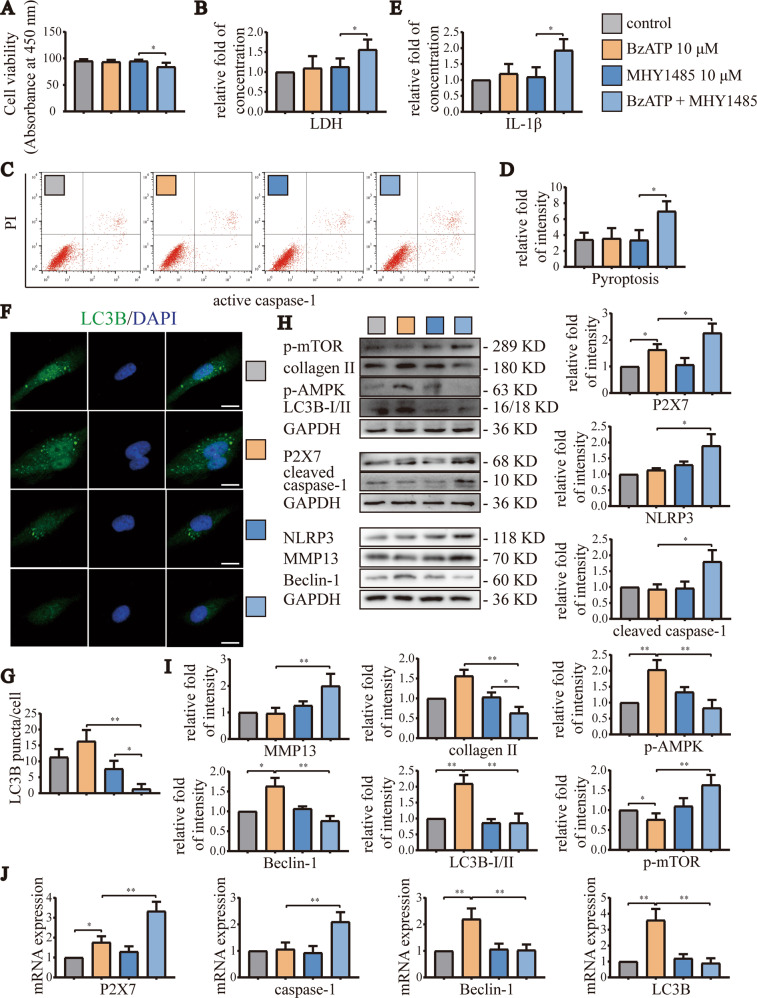
Moderate activation of P2X7 reduced pyroptosis through AMPK/mTOR-induced autophagy. **A** CCK-8 assays were used to evaluate cell viability in each group. Absorbance was measured at a wavelength of 450 nm. **B** LDH release assays were used to detect the degree of cell damage. **C** Flow cytometry was used to detect the number and ratio of caspase-1/PI-stained cells, reflecting the severity of cell pyroptosis. **D** Statistical data for stained cells. **E** ELISA was used to detect IL-1β content in cell culture supernatants for each group. **F** Cell fluorescence experiments were used to detect the fluorescence intensity and puncta number of cell LC3B staining, reflecting the level of autophagy (scale bar: 10 μm). **G** Statistical data for LC3B puncta/cell. **H**, **I** Western blotting and **J** RT-qPCR were used to detect protein and mRNA expression levels of P2X7, NLRP3, caspase-1, mTOR, AMPK, LC3B, Beclin-1, MMP13, and collagen II. Data are presented as means ± standard deviations of at least three independent experiments. *p < 0.05, **p < 0.01.

In order to explore whether appropriate activation of P2X7 promoted autophagy and thereby alleviated pyroptosis through the AMPK/mTOR signaling pathway, we treated cells with the mTOR activator MHY1485 (10 μM) and/or the AMPK inhibitor compound C (10 μM). CCK-8 assays (Fig. 6A) showed that application of MHY1485 alone did not cause significant changes in cell viability. However, when combined with BzATP (10 μM), cell viability decreased to 84.3% that of the control group. LDH release experiments (Fig. 6B) and flow cytometry analyses (Fig. 6C, D) showed that BzATP (10 μM) did not cause cell damage and pyroptosis. However, activation of mTOR resulted in increased LDH release and enhanced the ratio of caspase-1/PI-stained cells. Moreover, ELISA (Fig. 6E) also showed that inhibition of autophagy significantly increased IL-1β content in cell culture supernatants, indicating enhancement of pyroptosis. Cytofluorescence experiments (Fig. 6F, G) showed that LC3B aggregation was increased by treatment with BzATP (10 μM), but decreased after application of MHY1485, indicating that MHY1485 treatment blocked autophagy.

Western blotting (Fig. 6H, I) and RT-qPCR analyses (Fig. 6J) confirmed the above results. Briefly, treatment with BzATP (10 μM) increased the mRNA and protein expression levels of autophagy-related indicators. By contrast, MHY1485 treatment inhibited autophagy, increased the mRNA and protein expression levels of pyroptosis-related indicators, and enhanced the synthesis of MMP13 and the degradation of collagen II. Similar results were observed after treatment with compound C (Fig. S4). Taken together, these results suggested that moderate activation of P2X7 promoted autophagy through the AMPK/mTOR signaling pathway to alleviate pyroptosis. Activation of mTOR or inhibition of AMPK blocked autophagy and promoted pyroptosis, leading to aggravation of OA.

### Promotion of autophagy alleviated pyroptosis induced by overactivation of P2X7

**Fig. 7:**
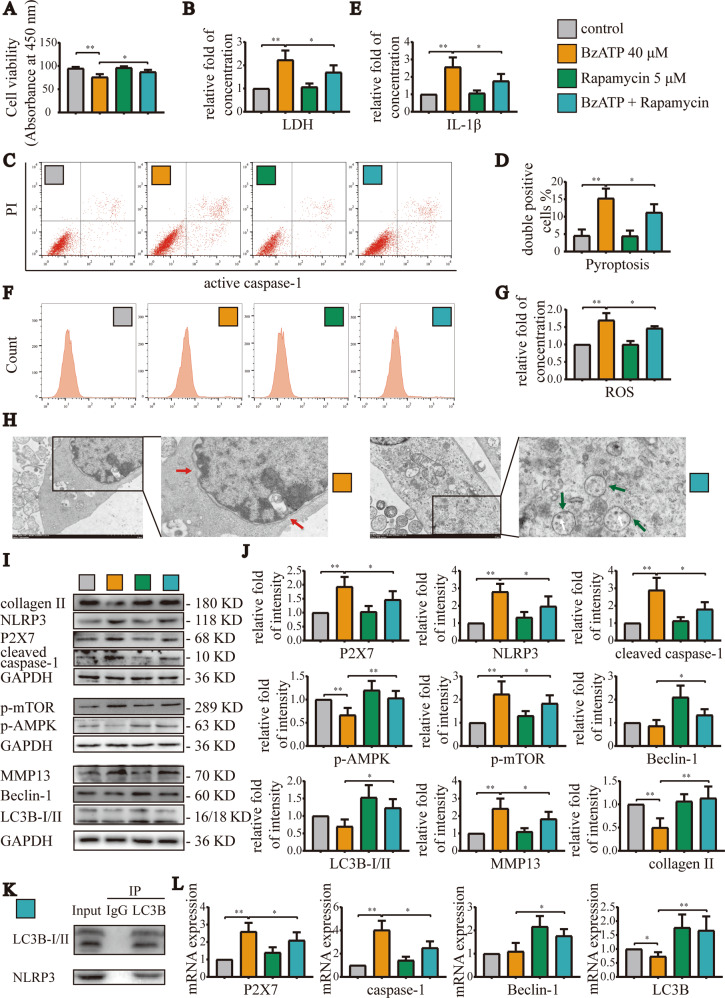
Increasing the level of autophagy effectively blocked pyroptosis induced by excessive activation of P2X7. **A** CCK-8 assays were used to detect cell viability for each group. Absorbance was measured at a wavelength of 450 nm. **B** LDH release assays were used to detect the degree of cell damage. **C**, **F** Flow cytometry was used to detect the number and ratio of caspase-1/PI- and ROS-stained cells, reflecting the severity of cell pyroptosis and ROS production. **D**, **G** Statistical data for stained cells. In the statistical analysis of ROS, we used the control group as a reference to normalize the absorbance value and expressed the results as the fold change. **E** ELISA was used to detect IL-1β content in cell culture supernatants for each group. **H** TEM analysis of cell morphology, including cell membrane rupture, nuclear membrane atrophy (red arrow), and autolysosome number (white arrow), reflecting the level of cell pyroptosis and autophagy (green arrow, autophagosome) (scale bar: 2 μm). **K** Co-IP experiments demonstrating the binding of LC3B and NLRP3. **I**, **J** Western blotting and **L** RT-qPCR were used to detect the protein and mRNA expression levels of P2X7, NLRP3, caspase-1, mTOR, AMPK, LC3B, Beclin-1, MMP13, and collagen II. Data are presented as means ± standard deviations of at least three independent experiments. *p < 0.05, **p < 0.01.

Based on our above experiments, we knew that decreasing autophagy would enhance pyroptosis. Therefore, in order to explore whether increasing the level of autophagy could alleviate the pyroptosis induced by P2X7 overactivation, we used the mTOR inhibitor rapamycin (5 μM) and the AMPK activator A-769662 (5 μM) for subsequent cell experiments. CCK-8 assays (Fig. 7A) showed that rapamycin alone did not cause significant changes in cell viability, but significantly restored cell viability after cotreatment with BzATP (40 μM). Moreover, LDH release assays (Fig. 7B) and flow cytometry showed that cell damage, ROS production (Fig. 7F, G), and pyroptosis (Fig. 7C, D) caused by BzATP (40 μM) were partial blocked by mTOR inhibition. Additionally, LDH release was reduced, and the caspase-1/PI and ROS staining ratio decreased. ELISA (Fig. 7E) also showed that the promotion of autophagy significantly decreased IL-1β content in cell culture supernatants, indicating that pyroptosis was partially blocked. The results of TEM (Fig. 7H) also confirmed that increased autophagy promoted the morphological integrity of the cells; the cell membrane remained intact, and the number of autophagic lysosomes increased.

Similar results were obtained by western blotting (Fig. 7I, J) and RT-qPCR (Fig. 7L). Indeed, treatment with BzATP (40 μM) increased in the mRNA and protein expression levels of pyroptosis-related indicators. However, treatment with rapamycin significantly decreased the expression levels of these targets, whereas the mRNA and protein expression levels of autophagy-related indicators increased. Additionally, treatment with rapamycin blocked the synthesis of MMP13 and the degradation of collagen II. Co-IP experiments (Fig. 7K) confirmed that LC3B could bind to NLRP3 and that autolysosomes could target and degrade inflammasomes to inhibit pyroptosis. Similar results were observed after treatment with A-769662 (Fig. S5). Collectively, these findings suggested that the promotion of autophagy increased the targeted degradation of inflammasome components by autolysosomes, thereby alleviating the pyroptosis induced by high-level activation of P2X7 and delaying the occurrence and development of OA.

## Discussion

**Fig. 8:**
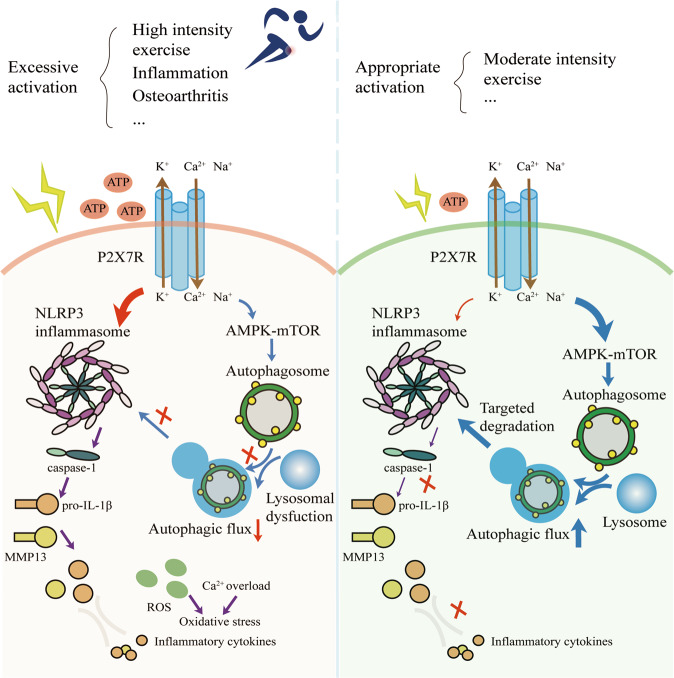
Mechanisms through which variations in P2X7 activation and expression affect autophagy and pyroptosis. Based on our findings, we established a model for the mechanisms through which P2X7 affects autophagy and pyroptosis. Briefly, when rats perform high-intensity exercise or chondrocytes receive high-intensity stimulation, P2X7 is overactivated, and the ion flow mediated by P2X7 activates the inflammasome pathway, causing the activation of caspase-1 and the release of active IL-1β. The AMPK/mTOR pathway mediated by P2X7 is insufficient to resist the damage caused by pyroptosis. By contrast, when rats perform moderate-intensity exercise or chondrocytes receive an appropriate intensity of stimulation, P2X7 is moderately activated, and the ion flow mediated by P2X7 mainly activates the AMPK/mTOR pathway, resulting in increased autophagy and enabling the induced autolysosomes to target the degradation of inflammasome components, thereby inhibiting pyroptosis and maintaining chondrocyte activity.

In this study, we found that under moderate-intensity exercise or stimulation with an appropriate concentration of BzATP, the activation state of P2X7 was low. Appropriate activation of P2X7 promoted autophagy through the AMPK/mTOR signaling pathway, inhibited pyroptosis, and then blocked the occurrence and development of OA. By contrast, overactivation of P2X7, such as that occurring by stimulation with high-intensity exercise or a high concentration of BzATP, increased mTOR activation and decreased autophagy. These effects were not sufficient to counteract the massive activation of NLRP3 inflammasomes, resulting in pyroptosis, cell death, and cartilage degradation (Fig. 8).

As an important signaling pathway downstream of P2X7, AMPK signaling participates in the regulation of many physiological and disease functions [24], such as the promotion of autophagy by exercise [25]. During exercise, the interaction between the innate immune molecule Toll-like receptor 9 and Beclin-1 is strengthened to regulate AMPK activation in muscles [26]. AMPK interacts with sestrins to participate in exercise-induced autophagy and thereby maintains skeletal muscle glucose metabolism [27]. However, another study showed that autophagy plays important role in maintaining mitochondrial function during exercise, but has little effect on PRKAA1/AMPK activation, exercise-dependent glucose homeostasis, and energy supply to satisfy muscle contraction [28]. Despite these inconsistencies, the effects of AMPK activation-induced autophagy on alleviating inflammation are unquestionable. Post-exercise pretreatment (10 min rest for every 10 min running) promotes autophagy through AMPK/mTOR signaling to alleviate heart damage caused by exhaustive exercise [29], and downregulation of autophagy promotes accumulation of damaged mitochondria, increases ROS, and leads to tissue degeneration [30]. Our experimental results also showed that AMPK expression was higher in the moderate-intensity exercise group than in the low- and high-intensity exercise groups; mTOR expression showed the opposite trend. Thus, taken together with our findings of changes in the expression levels of autophagy- and pyroptosis-related indicators, we concluded that moderate-intensity exercise decreased P2X7 expression and activity, promoted autophagy, and inhibited pyroptosis. However, further studies are needed to clarify the involvement of P2X7 and the AMPK/mTOR signaling pathway in this mechanism.

As a key effector of inflammation [31], P2X7 mediates multiple signaling pathways to modulate autophagy, pyroptosis, metabolism, and nutrition [32]. Compared with other P2X receptors, P2X7 requires a higher concentration of ATP for activation [33] and has a higher affinity for the selective agonist BzATP, whose potency is dozens of times that of ATP [4]. Our cell experiments showed that as the concentration of BzATP increased, the expression of P2X7 increased. Notably, the P2X7-mediated ion current not only induces autophagy but also promotes pyroptosis. Moreover, during the early stages of activation, the P2X7-mediated cellular response is dominated by autophagy; in the later stages of activation, the main phenotype of the cell gradually changes from autophagy to pyroptosis. This finding is similar to the results of another study demonstrating that initial activation of P2X7 has protective effects on cells but as the activation time is increased, cell death is induced [19]. Ca^2+^ overload, caused by P2X7R-mediated Ca^2+^ influx under stimulation with high concentrations of ATP in macrophages, leads to mitochondrial dysfunction, which in turn causes cell pyroptosis. However, when P2X7R was stimulated with low ATP concentrations or was positively allosterically regulated by compound K, the accumulation of mtROS and the activation of caspase-1 and -3 promoted apoptosis rather than pyroptosis as the mechanism of cell death [34].

The activation level of P2X7 is closely related to the direction of cell fate. Regulation of the AMPK/mTOR signaling pathway by P2X7 promotes autophagy, exerts antitumor effects [35], and protects against *Mycobacterium tuberculosis* infection in macrophages [36]. However, some studies have reported conflicting results. For example, P2X7 activates the phosphatidylinositol 3-kinase/AKT/mTOR pathway and other signaling pathways to promote the proliferation and metastasis of osteosarcoma and increase bone destruction [37]. Additionally, P2X7 expression is downregulated in an inflammatory environment, and its activation promotes the osteogenesis of periodontal ligament stem cells [38]. In our study, treatment with AMPK and mTOR activators or inhibitors showed that the AMPK/mTOR pathway was a key signaling pathway through which P2X7 induced autophagy. Inhibition of AMPK or activation of mTOR blocked the autophagy-inducing effects of P2X7, even if P2X7 was only moderately activated, and insufficient autophagy would then lead to aggravation of pyroptosis. However, the link between P2X7-induced autophagy and pyroptosis remains unknown.

When cells undergo pyroptosis, autophagy usually plays protective roles. In the resting state of macrophages, autophagy is inhibited. However, in the presence of a low degree of infection, inhibition of autophagy is released, resulting in cell protection; when autophagy cannot eliminate intracellular infection, caspase-1 is activated to initiate pyroptosis [39]. Autophagy often suppresses pyroptosis through the fusion of autophagosomes and lysosomes to form autolysosomes, which further degrade intracellular harmful substances, such as unfolded proteins and damaged mitochondria [40], as well as inflammasome components, such as NLRP3 [41] and ASC [42]. The interaction between LC3B and NLRP3 is also essential for autophagy-mediated degradation of NLRP3 and inhibition of pyroptosis [43]. In this study, we verified the interaction between LC3B and NLRP3 in chondrocytes through co-IP experiments and found that proper activation P2X7-induced autophagy inhibited pyroptosis by degrading NLRP3. In this study, we showed that AMPK activators or mTOR inhibitors effectively increased autophagy and significantly alleviated pyroptosis and inflammation caused by excessive activation of P2X7. Based on the above results, we concluded that regulation of the AMPK/mTOR signaling pathway by P2X7 played important role in inducing autophagy and inhibiting pyroptosis.

In this study, we demonstrated the role of P2X7 in the prevention and treatment of OA by exercise for the first time. Moderate activation of P2X7 can effectively relieve inflammation, providing insights into novel approaches for OA treatment. However, further studies are still required. For example, cell models involving stress and stretching [44] may be more consistent with the in vivo environment under exercise conditions. Therefore, in subsequent studies, we will evaluate the effects of P2X7 expression on chondrocytes under different degrees of mechanical stress stimulation.

## Conclusion

In summary, we explored the expression and activation of P2X7 under different intensities of exercise or stimulation and confirmed that the appropriate intensity of exercise or moderate stimulation led to activation of P2X7 to a certain level, exerting protective effects on cells. The AMPK/mTOR signaling pathway was found to play an indispensable role in P2X7-induced autophagy, and the formation of autolysosomes affected the degradation of inflammasome components and reduced cell damage. We also confirmed, for the first time, that moderate-intensity exercise promoted autophagy through the P2X7/AMPK/mTOR signaling axis, inhibited pyroptosis, and thereby alleviated OA. These findings regarding the role of P2X7 in exercise-based prevention and treatment of OA provide new perspectives for OA treatment.

## Materials and methods

### Antibodies and reagents

The antibodies used in this study were as follows: anti-P2X7 (Abcam, Cambridge, UK; cat. no. ab109054), anti-collagen II (Abcam; cat. no. ab34712), anti-MMP13 (Abcam; cat. no. ab39012), anti-AMPKα1 (Abcam; cat. no. ab32047), anti-mTOR (Abcam; cat. no. ab109268), anti-NLRP3 (Proteintech; cat. no. 19771-1-AP), anti-caspase-1 (Proteintech; cat. no. 22915-1-AP), anti-LC3B (Abcam; cat. no. ab192890), anti-Beclin-1 (Abcam; cat. no. ab62557), anti-glyceraldehyde 3-phosphate dehydrogenase (GAPDH; Proteintech; cat. no. 10494-1-AP), and horseradish peroxidase (HRP)-labeled IgG (Beyotime; cat. no. A0208). The reagents used in the experiment were as follows: MIA (Sigma, St. Louis, MO, USA; cat. no. I2512), the P2X7 receptor agonist BzATP (Sigma; cat. no. B6396), the mTOR activator MHY1485 (Sigma; cat. no. SML0810), the mTOR inhibitor rapamycin (Sigma; cat. no. V900930), the NLRP3 inhibitor CY-09 (Sigma; cat. no. SML2465), the AMPK activator A-769662 (Sigma; cat. no. SML2578), and the AMPK inhibitor compound C (Sigma; cat. no. P5499). The concentrations, dosages, and preparation of the reagents were described previously [43, 45].

### Animal models and extraction of rat tissue

Fifty Sprague-Dawley (SD) rats (male, 5 weeks old, 230–250 g) were purchased from HFK Bioscience (Beijing, China) and were randomly assigned into the following five groups (*n* = 10 each; Fig. S1): [1] control group (normal saline) [2], OA group (MIA)[3], exercise group (MIA + treadmill exercise) [4], agonist group (MIA + treadmill exercise + BzATP), and [5] inhibitor group (MIA + treadmill exercise + MHY1485 [an autophagy inhibitor]). We considered that male rats are more capable of running on a treadmill, plus the previous research results of our research group, so we chose male SD rats for animal experiments. Under the premise of ensuring sufficient data, we used as few animals as possible to conduct experiments. In the end, we used a total of 50 SD rats, which were randomly allocated, and 10 in each group were used for subsequent animal experiments. Animal-related experiments complied with the Animal Ethics Regulations of China Medical University (approval no. 2017PS237K) and were run in blinded fashion. During the experiments, we adhered to the 3R rules to ensure that rats were sacrificed comfortably.

MIA, BzATP, and MHY1485 were prepared as 50 μL aliquots and were injected into the knee joint cavity of rats after 3 days of adaptive feeding. The control group was injected with the same volume of normal saline, and the other four groups were injected with 0.5 mg MIA for each knee joint (only once); the agonist and inhibitor groups were also injected with 0.3 mg BzATP or MHY1485 (twice a week). The dosage of the reagent and the frequency of injection referred to the results of our previous research [20, 23] and the results of our pre-experiment. For 5 weeks, the first two groups of rats remained sedentary, and the other three groups performed adaptive treadmill exercise for 1 week and then maintained appropriate intensity exercise for 4 weeks (Monday through Friday, 1 h/day, treadmill speed of 18 m/min). After modeling, the rats were anesthetized and euthanized with 1.5% pentobarbital sodium 0.2 mL/100 g, intraperitoneal injection, and the intra-articular lavage fluid (IALF) was collected immediately for enzyme-linked immunosorbent assays (ELISAs). Part of the knee joint tissue was subjected to micro-computed tomography (micro-CT) analysis, and the remaining tissue was used for subsequent experiments.

### Histological analysis and immunohistochemistry (IHC)

The removed knee joint tissue was fixed with 4% paraformaldehyde for 48–72 h, decalcified with 10% ethylenediaminetetraacetic acid-2Na for 4–6 weeks, and embedded in paraffin. An automatic staining machine (Leica) was used for hematoxylin and eosin (H&E) staining, and cartilage staining solution (Solarbio; cat. no. G2543) was used for Toluidine Blue O staining. After deparaffinization, antigen retrieval (Boster; cat. no. AR0026) was performed, and a universal two-step detection kit (ZSGB-BIO; cat. no. PV-9000) was used to remove peroxidase according to the manufacturer’s instructions. Sections were then blocked with goat serum, incubated with primary antibodies overnight at 4 °C followed by incubation with secondary antibodies, and subjected to diaminobenzidine staining and hematoxylin staining. After staining and IHC, the sections were dehydrated, mounted with neutral gum, and photographed with an Eclipse E800 fluorescence microscope (Nikon, Tokyo, Japan).

The same approach was used for terminal deoxynucleotidyl transferase dUTP nick-end labeling (TUNEL) assays. The reason we chose the TUNEL experiment to detect the degree of cell death was that the dead chondrocytes in the paraffin section can be combined with TUNEL staining reagents to present a unique luminescence phenomenon under a fluorescence microscope, and the degree of chondrocyte death can be clearly and easily observed. Briefly, after antigen retrieval, the slices were permeabilized with 0.1% Triton X-100 at room temperature for 10–30 min and then incubated with the reaction mixture overnight at 4 °C. The next day, sections were counterstained with 4′,6-diamidino-2-phenylindole (DAPI) and then recorded with a fluorescence microscope (Nikon; cat. no. E800). When we conducted the statistical analysis, we collected data from at least six animals in each group, and at least five images of each animal were selected for analysis.

### Micro-CT

The collected knee joint tissue was fixed with 4% paraformaldehyde for 24–48 h, rinsed once with 75% alcohol, soaked in 75% alcohol, stored at 4 °C, and tested immediately. A Skyscan 1276 (Bruker, Kontich, Belgium) and NRecon version 1.6 were used to analyze the proximal tibia and subchondral bone area. Bone volume (BV), BV/total tissue volume (BV/TV), trabecular number (Tb.N), trabecular thickness (Tb.Th), and trabecular separation (Tb.Sp) were determined. CTAn version 1.9 was used for data analysis.

### Primary chondrocyte culture

Phenobarbital was used to anesthetize and euthanize male SD rats (4 weeks old). Cartilage tissue was removed from the femoral head and distal femur in a sterile environment. Next, protease K (30 min, 4 mg/mL; Roche) was used to digest the chopped cartilage tissue at 37 °C for 1–2 h. Samples were then treated with collagenase D (2 h, 1.6 mg/mL; Roche) for additional digestion for 4–6 h. The obtained cells were plated in T25 culture flasks at an appropriate density and cultured in Dulbecco’s modified Eagle’s medium/F12 medium (Gibco, Thermo Fisher Scientific) containing 10% fetal bovine serum and 1% penicillin/streptomycin at 37 °C in an atmosphere containing 5% CO_2_ for 5–7 days. Cells were then passaged. Second- or third-generation cells were used in subsequent experiments.

In cell experiments, the reagents we used include P2X7 agonist BzATP, NLRP3 inhibitor CY-09 (10 μM), AMPK agonist A769662 (5 μM) and inhibitor Compoud C (10 μM), mTOR agonist MHY1485 (μM), and inhibitor rapamycin (5 μM). The dosage and treatment time of BzATP referred to our previous research results [11, 20] and subsequent cell experiment results. The gradient was set to 10, 20, 30, and 40 μM, and the treatment time was 12 h. The processing time of other reagents was the same as that of BzATP, which was 12 h. The dosage of other reagents was selected according to our previous research results, reagent instructions, and the results of preliminary experiments. After determining the appropriate dosage and processing time of the above reagents, they can be used in subsequent cell experiments.

### Cell viability assays and lactate dehydrogenase (LDH) release tests

The cells were seeded in 96-well plates at a density of 5000 cells/well, and 200 μL culture medium was added to each well. Cells were then incubated for 24–48 h, and appropriate reagents were added. After the reaction was completed, 90 μL culture medium and 10 μL Cell Counting Kit (CCK)-8 detection solution (Beyotime; cat. no. C0042) were added to each well. Cells were incubated for an additional 1–2 h and then placed in a microplate reader (Synergy H1; BioTek, USA) to detect luminescence at a wavelength of 450 nm.

The same approach was used for LDH release experiments. When the cell confluence reached 80–90%, appropriate drug treatments were applied. One hour before the detection time point, LDH release reagent provided by the kit (Beyotime; cat. no. C0017) was added to the sample maximum enzyme activity control well at 10% of the original culture medium volume. Samples were mixed by pipetting several times and then further incubated in the cell culture incubator. After reaching the predetermined time, the cell culture plates were centrifuged using a multi-well plate centrifuge at 400 × *g* for 5 min. Finally, 120 μL of the supernatant from each well was added to the corresponding well of a new 96-well plate, and sample determination was performed to measure absorbance at 590 and 680 nm using a Synergy H1 microplate reader (BioTek Instruments). The fold increase in LDH concentration was normalized to the control.

### ELISA

All reagents, standard dilutions, and samples (cell supernatants and IALF) were prepared using a Rat IL-1β/IL-1F2 Quantikine ELISA Kit (R&D Systems; cat. no. RLB00) according to the manufacturer’s instructions. Samples were washed with phosphate-buffered saline (PBS) between each step. Briefly, 50 µL analytical diluent was added to each well, and 50 µL standard solution, control solution, or sample was added to each well. Plates were incubated for 2 h at room temperature, 100 µL conjugate was added to each well, and plates were incubated for an additional 2 h at room temperature. Next, 100 µL substrate solution was added to each well, plates were incubated at room temperature for 30 min in the dark, and 100 µL stop solution was added to each well. Finally, the absorbance was read at 450 nm within 30 min, with the wavelength correction set to 540 or 570 nm.

### Flow cytometry

After appropriate treatment, cells were collected by trypsin digestion and centrifuged at 300 × *g* for 5 min. Next, caspase-1 FLICA (660-YVAD-FMK) was added, and cells were incubated at 37 °C for 1 h and stained with propidium iodide (PI) for 15 min. Fluorescence-activated cell sorting was then performed using a FACSCalibur instrument (BD Biosciences, USA) to detect the corresponding emission wavelength. Flow Jo software (BD Biosciences) was used for data analysis.

### Western blotting

Treated cells were lysed with radioimmunoprecipitation assay (RIPA) lysis buffer (Beyotime, P0013B) containing protease/phosphatase inhibitors, placed on ice for 20 min, and then centrifuged at 14000 × *g* for 20 min. The supernatants were removed, and a bicinchoninic acid protein assay kit (Beyotime; cat. no. P0010) was used to measure protein concentrations. Next, an appropriate volume of 5× loading buffer was added, and protein samples were boiled to denature the protein and stored at −20°C until use. A PAGE Gel Fast Preparation Kit (Epizyme; cat. no. PG112) was used to prepare gels, and protein samples were then electrophoresed and transferred to membranes. Membranes were blocked with 5% skim milk at room temperature for 2 h and incubated with primary antibodies (1:1000) overnight at 4 °C. Next, membranes were incubated with HRP-labeled IgG antibodies (1:2000) at room temperature for 2 h and scanned using a chemiluminescence detection system (GE Amersham Imager 600). Image J (NIH, USA) was used to analyze the gray values of the bands, and GAPDH was used as an internal control. Statistical analysis was performed after the values were normalized to GAPDH expression.

### RNA isolation and reverse transcription-quantitative polymerase chain reaction (RT-qPCR)

First, 1 mL TRIzol (TaKaRa, Shiga, Japan; cat. no. 9109) was added to each well of a six-well plate containing treated cells. RNA was then isolated and purified according to the manufacturer’s instructions. A Nanophotometer 50 instrument was used to measure RNA concentrations and purity, and the A260/A280 values of all samples were between 1.8 and 2.0, indicating high RNA quality. Prime Script RT Master Mix (TaKaRa; cat. no. RR036A) was used for reverse transcription, and TB Green Premix Ex Tag II (TaKaRa; cat. no. RR820A) was used for amplification on a 7500 Real-Time PCR System (Applied Biosystems, Foster City, CA, USA). Thermocycling conditions were as follows: 95 °C for 30 s, followed by 40 cycles at 95 °C for 5 s and 60 °C for 35 s. The obtained Ct values were normalized using the 2^−ΔΔCt^ formula, with *GAPDH* as the housekeeping gene. The primer sequences are detailed in Supplementary Fig. S2.

### Immunofluorescence and imaging analysis

Treated cells were fixed with 4% paraformaldehyde for 10 min, permeabilized with 0.1–0.2% Triton X-100 for 10 min, and then blocked with 5–10% serum for 30 min. Cells were then incubated with primary antibodies (1:100) overnight at 4 °C, followed by incubation with secondary antibodies (1:200) for 1 h at room temperature in the dark. Finally, cells were incubated with DAPI to stain nuclei at room temperature for 5 min in the dark. A Zeiss LSM880 confocal microscope (Zeiss, Oberkochen, Germany) was used to image the cells, and the images were analyzed using Image J.

### Co-immunoprecipitation (Co-IP) assay

RIPA was added to lyse the cells, and 600 μL protein sample (600 μg protein) was added to 1 μg ordinary IgG and 20 μL resuspended Protein G Agarose. Samples were shaken slowly at 4 °C for 30 min to 2 h and centrifuged at 1000 × *g* for 5 min. The supernatants were then used for subsequent immunoprecipitation. Briefly, 0.2–2 μg primary antibody was added, and samples were shaken slowly overnight at 4 °C. Next, 20 μL fully resuspended Protein G Agarose was added, and samples were shaken slowly at 4 °C for 1–3 h. Samples were centrifuged at 1000 × *g* for 5 min, supernatants were removed, pellets were washed with PBS, the supernatants were removed again, and 20–40 μL 1 × sodium dodecyl sulfate-polyacrylamide gel electrophoresis (SDS-PAGE) loading buffer was added. Samples were vortexed to resuspend the pellet and then centrifuged. Finally, samples were boiled at 100 °C for 10 min and subjected to SDS-PAGE.

### Transmission electron microscopy (TEM)

Cultured cells were treated with 2.5% normal temperature glutaraldehyde fixative after removal of culture medium and fixed for 5 min at room temperature in the dark. A cell scraper was used to gently remove the cells, and the cell liquid was moved to a centrifuge tube and centrifuged at 1000 × *g* for 2 min. After discarding the fixative, 1% OsO_4_ was added, and the cell cluster was gently lifted and suspended in the fixative. Cells were fixed for 30 min at room temperature in the dark and then transferred to 4 °C until analysis. After dehydration, samples were embedded in resin. Ultrathin tissue sections (60 nm) were stained with uranyl acetate and lead citrate, and the cell morphology and subcellular structure were then observed using TEM (Hitachi 800; Hitachi, Tokyo, Japan).

### Statistical analysis

We performed at least three independent experiments, and the average values of each experimental group were used in the statistical analysis. Data are expressed as means ± standard deviations. The between-group differences were determined using Student’s *t*-test or one-way analysis of variance with Tukey’s post hoc test in GraphPad Prism version 7.0c. Significance was set at *p* < 0.05.

## Supplementary information

Supplementary_Material

## Data Availability

All original data are available from the corresponding author upon request. Source data for Figs. 4–7 are provided with the paper.
