# Supplementary material for: Moderate-intensity exercise alleviates pyroptosis by promoting autophagy in osteoarthritis via the P2X7/AMPK/mTOR axis
Source: Cell Death Discov. 2021 Nov 10;7:346. doi: 10.1038/s41420-021-00746-z (PMC8580998; doi:10.1038/s41420-021-00746-z)
Supplement: Supplementary file 1 — Supplementary_Material [file 41420_2021_746_MOESM1_ESM.docx]

Supplementary Material

# Figure S1: Design of the treatment schedule


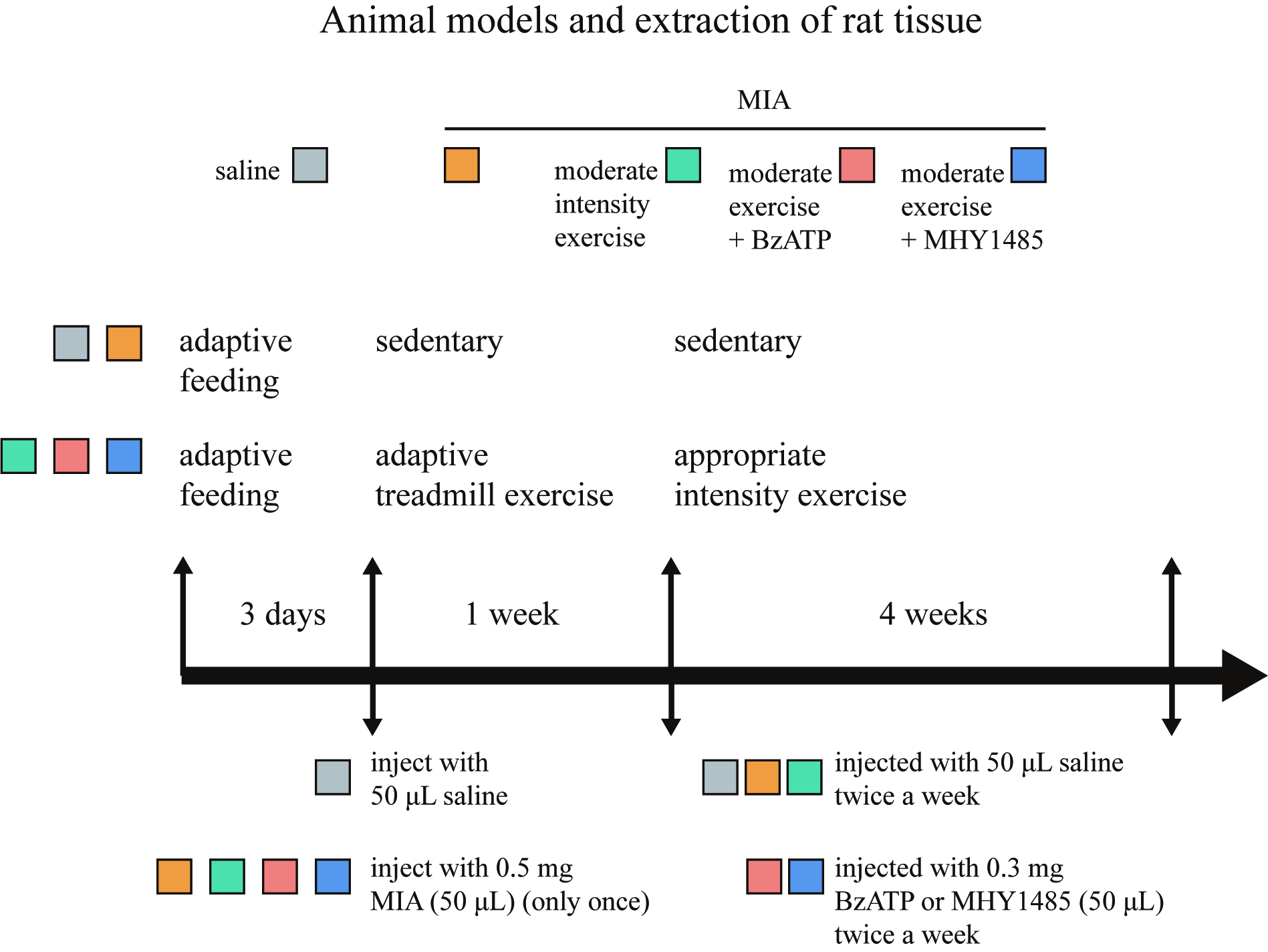


# Figure S2: Rat primer sequences

| Gene | Species |  | Sequence |
| --- | --- | --- | --- |
| *P2X7* | rat | forward | 5′- TTAGTACACGGCATCTTCGACACG - 3′ |
|  |  | reverse | 5′- AGCTTCTGTTCTTGGCCTTCTGAC - 3′ |
| *MMP13* | rat | forward | 5′- TGGTCCAGGAGATGAAGACC - 3′ |
|  |  | reverse | 5′- GTGCAGACGCCAGAAGAATC - 3′ |
| Collagen II | rat | forward | 5′- ACGCTCAAGTCGCTGAACAACC -3′ |
|  |  | reverse | 5′- ATCCAGTAGTCTCCGCTCTTCCAC -3′ |
| Beclin-1 | rat | forward | 5′- CTGGACCGAGTGACCATTCA - 3′ |
|  |  | reverse | 5′- AGACACCATCCTGGCGAGTT - 3′ |
| *LC3B* | rat | forward | 5′- GTCGCTAACAAGCAGTGGGA - 3′ |
|  |  | reverse | 5′- AGGGCTTCTGGGGCTCTAAT - 3′ |
| Caspase-1 | rat | forward | 5′- TTTCCGCAAGGTTCGATTTTCA - 3′ |
|  |  | reverse | 5’- GGCATCTGCGCTCTACCATC - 3’ |
| *GAPDH* | rat | forward | 5′- GGCACAGTCAAGGCTGAGAATG - 3′ |
|  |  | reverse | 5′- ATGGTGGTGAAGACGCCAGTA - 3′ |

# Figure S3: Unmerged images from Figures 1A and 3A


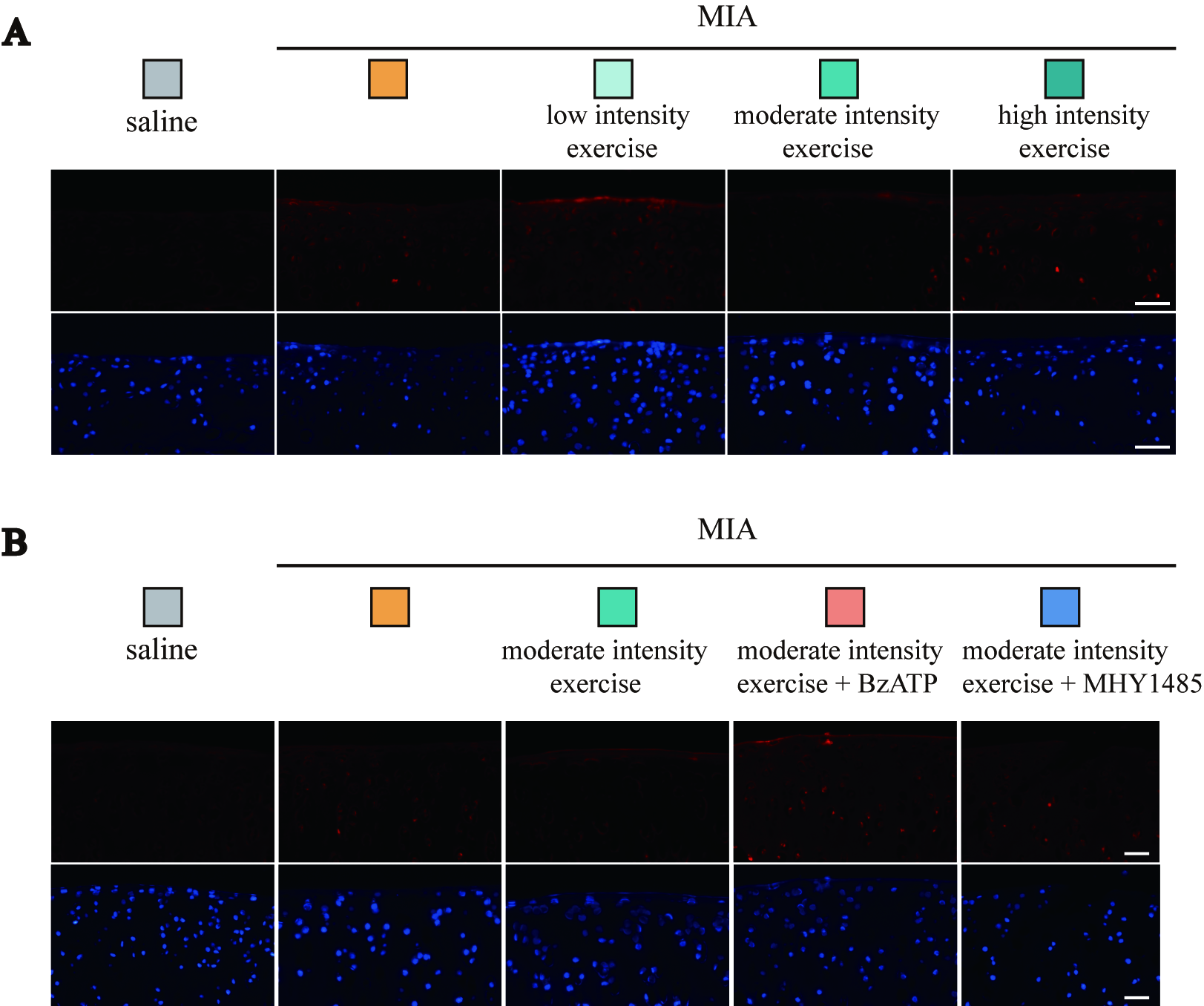


Figure S3: (A) TUNEL assays (Figure 1A) showed that moderate-intensity exercise significantly reduced cell death in the knee joint tissue of OA model rats compared with low- and high-intensity exercise. (B) BzATP upregulated P2X7, and MHY1485 inhibited autophagy; thus, the anti-inflammatory effects were blocked. Additional injection of these compounds significantly increased numbers of dead cells (Figure 3A).

# Figure S4: Supplement to Figure 6

#
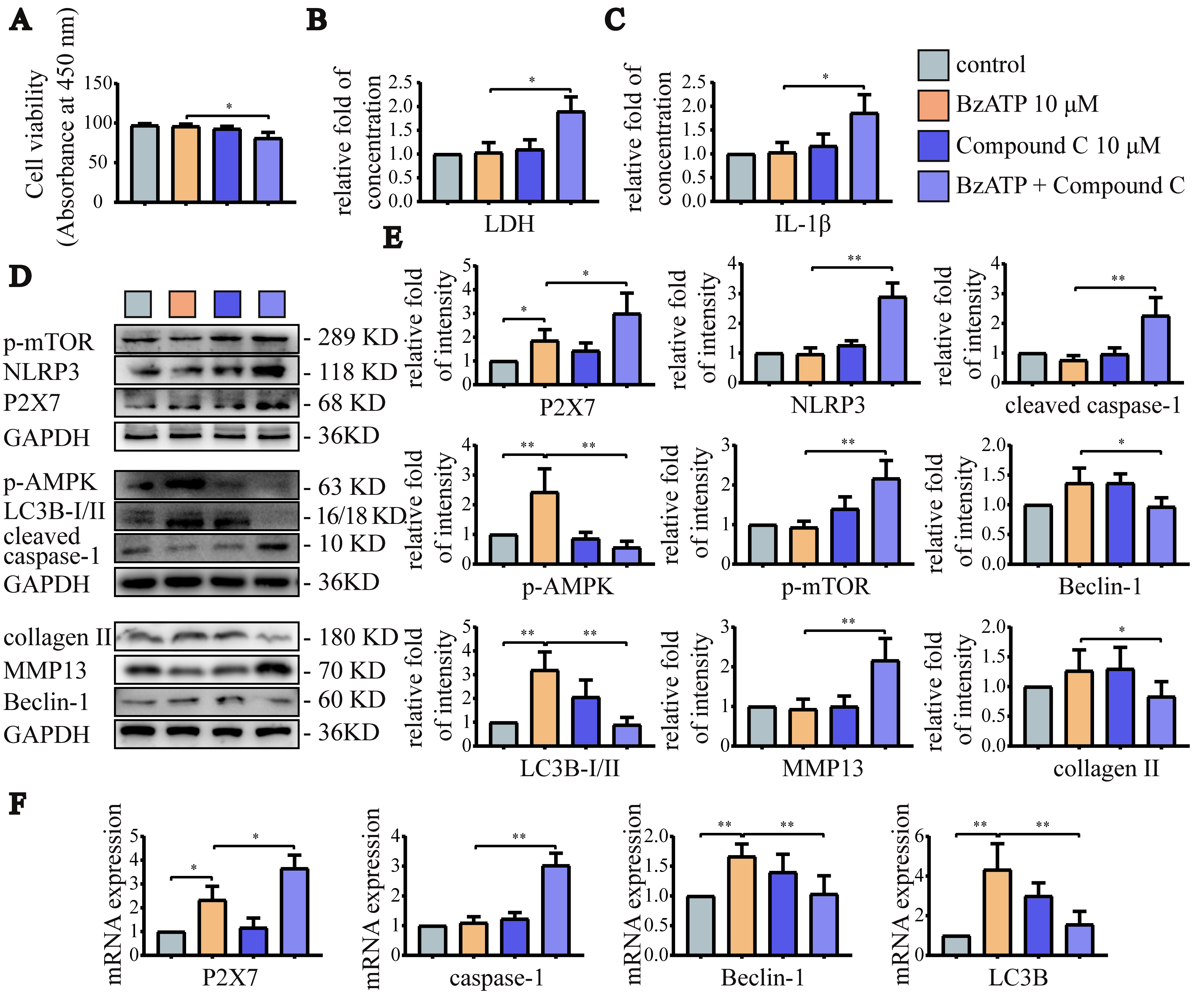


Figure S4: (A) CCK-8 assays were used to detect cell viability in each group. Absorbance was measured at 450 nm. (B) LDH release assays were used to detect the degree of cell damage. (C) ELISA was used to determine IL-1β content in cell culture supernatants for each group. (D, E) Western blotting and (F) RT-qPCR analyses were used to detect the protein and mRNA expression levels of P2X7, NLRP3, caspase-1, mTOR, AMPK, LC3B, Beclin-1, MMP13, and collagen II. Data are presented as means ± standard deviations of at least three independent experiments. ^⁎^*p* < 0.05, ^⁎⁎^*p* < 0.01.

# Figure S5: Supplement to Figure 7

#
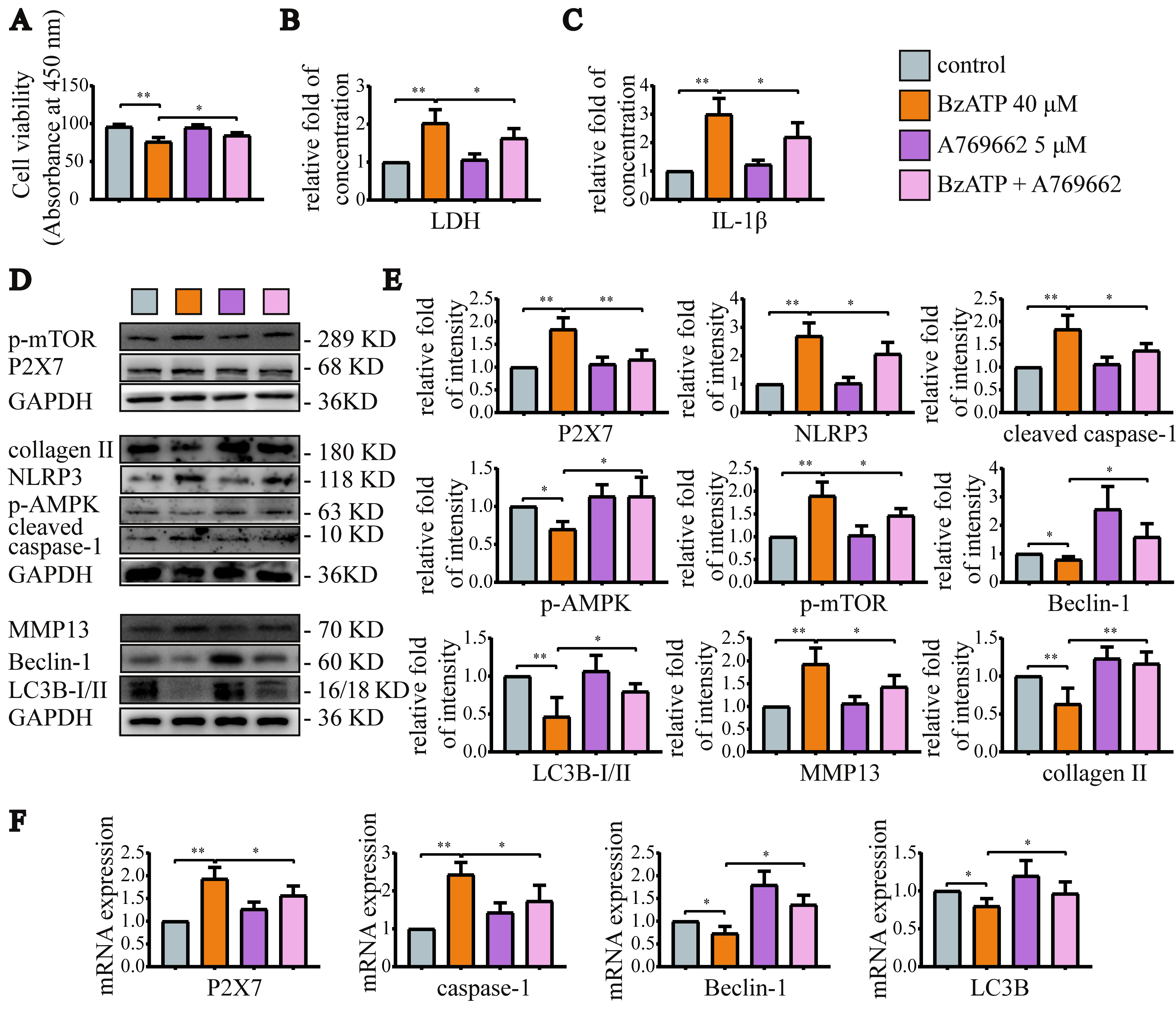


Figure S5: (A) CCK-8 assays were used to determine cell viability in each group. Absorbance was measured at a wavelength of 450 nm. (B) LDH release assays were used to detect the degree of cell damage. (C) ELISA was used to determine IL-1β content in cell culture supernatants for each group. (D, E) Western blotting and (F) RT-qPCR were used to detect the protein and mRNA expression levels of P2X7, NLRP3, caspase-1, mTOR, AMPK, LC3B, Beclin-1, MMP13, and collagen II. Data are presented as means ± standard deviations of at least three independent experiments. ^⁎^*p* < 0.05, ^⁎⁎^*p* < 0.01.

# Supplement to “Animal models and extraction of rat tissue”

Our criteria for excluding animals are determined based on expected experimental results and experience, such as model failure: the knee joint cavity cannot be injected with drugs or severe infections in the knee joint cavity, such as poor health of rats: pulmonary edema, fractures and other diseases. We number 50 rats corresponding to 1-50, and then use computer software to randomly group them into 5 groups, each with 10 rats, so as to achieve the purpose of random grouping.

Regarding the creation of the OA rat model, first, we anesthetized the rat with an appropriate amount of phenobarbital (1.5% pentobarbital sodium 0.2 mL/100 g) in a sterile environment. Next, the hair on the knee joints of the rats was shaved. Subsequently, use a 50-μL injection needle to inject the well-proportioned MIA solution (0.5 mg MIA dissolved in 50-μL normal saline) along the inside of the knee joint cavity. During the injection, the knee joints of the rats were properly moved to ensure that the MIA solution was evenly distributed in the joint cavity. Finally, disinfected the injection site. Throughout the experimental period, only one injection of MIA solution can complete the creation of OA rat model.

In order to reduce the harm to animals, we tried to choose a small reagent dose and injection frequency under the premise of achieving experimental results. After optimization, we found that 0.5 mg of MIA can create a rat OA model, and 0.3 mg of BzATP and MHY1485 can achieve the effect of increasing the expression levels of P2X7 and mTOR. Therefore, we chose the above dosage and injection frequency, and dissolved the reagent in 50-μL of normal saline for animal experiments.

# Supplement to “Histological analysis and immunohistochemistry (IHC)”

The time for tissue fixation and decalcification was selected based on the laboratory's operating experience and reagent instructions. 4% paraformaldehyde treatment for 2-3 days was sufficient to fix the tissue. The decalcification solution was changed twice a week, usually 4-6 weeks was enough to soften the knee joint, which was convenient for embedding and sectioning in subsequent steps.

# Supplement to “Western blotting”

We used a 10% gel for protein electrophoresis. This concentration gel can cover proteins with a molecular weight of 10 - 300 KD. The electrophoresis time of the upper gel (concentrated gel) was 30 min and the voltage was 80 V, and the electrophoresis time of the lower gel (separation gel) was 90 min and the voltage was 120 V. This was to ensure that the distance between proteins of various molecular weights is large enough. Coupled with the loading marker (Multicolor Prestained Protein Ladder) (Epizyme; cat. no. WJ102), after the electrophoresis was completed, the marker marks of various molecular weights can be clearly observed, which facilitates the subsequent gel cutting step. In the process of gel cutting, we cut off the gel with the required molecular weight range according to the marker marks on both sides of the lane. According to the principle of two points to determine a straight line, cut the gel containing the target protein into a rectangle. The sandwich structure (filter paper/gel/band/filter paper) was then adopted for the subsequent transfer membrane steps. We used 200 mA constant current to transfer the membrane, the transfer time was 50 min for the bands in the molecular weight range of 10 - 50 KD, and the transfer time was 80 min for the bands in the 50 - 300 KD molecular weight range.

# Supplement to “Statistical analysis”

Before statistical analysis, we first judged whether the data conformed to a normal distribution or homogeneity of variance by using GraphPad Prism version 7.0c software. We commonly use Shapiro-Wilk (SW) tests and Kolmogorov-Smirnov (KS) tests. It is generally believed that when the *p* value is greater than 0.1, the data obey a normal distribution. We then performed subsequent statistical analyses based on the results.
